# Clinical Response of Major Depressive Disorder Patients With Suicidal Ideation to Individual Target-Transcranial Magnetic Stimulation

**DOI:** 10.3389/fpsyt.2021.768819

**Published:** 2021-11-05

**Authors:** Nailong Tang, Chuanzhu Sun, Yangtao Wang, Xiang Li, Junchang Liu, Yihuan Chen, Liang Sun, Yang Rao, Sanzhong Li, Shun Qi, Huaning Wang

**Affiliations:** ^1^Department of Psychiatry, Xijing Hospital, Fourth Military Medical University, Xi'an, China; ^2^Department of Psychiatry, 907 Hospital of Joint Logistics Team, Nanping, China; ^3^Brain Modulation and Scientific Research Center, Xi'an, China; ^4^The Key Laboratory of Biomedical Information Engineering, Ministry of Education, Department of Biomedical Engineering, School of Life Science and Technology, Xi'an Jiaotong University, Xi'an, China; ^5^The Key Laboratory of Biomedical Information Engineering of Ministry of Education, Institute of Health and Rehabilitation Science, School of Life Science and Technology, Xi'an Jiaotong University, Xi'an, China; ^6^Department of Neurosurgery, Xijing Hospital, Fourth Military Medical University, Xi'an, China; ^7^Neuromodulation Lab of Brain Science and Humanoid Intelligence Research Center, Xi'an Jiaotong University, Xi'an, China

**Keywords:** individual target-transcranial magnetic stimulation, major depressive disorder, suicidal ideation, functional network connectivity, independent component analysis

## Abstract

Suicidal ideation increases precipitously in patients with depression, contributing to the risk of suicidal attempts. Despite the recent advancement in transcranial magnetic stimulation, its effectiveness in depression disorder and its wide acceptance, the network mechanisms of the clinical response to suicidal ideation in major depressive disorder remain unclear. Independent component analysis for neuroimaging data allows the identification of functional network connectivity which may help to explore the neural basis of suicidal ideation in major depressive disorder. Resting-state functional magnetic resonance imaging data and clinical scales were collected from 30 participants (15 major depressive patients with suicidal ideation and 15 healthy subjects). Individual target-transcranial magnetic stimulation (IT-TMS) was then used to decrease the subgenual anterior cingulate cortex activity through the left dorsolateral prefrontal cortex. Thirty days post IT-TMS therapy, seven of 15 patients (46.67%) met suicidal remission criteria, and 12 patients (80.00%) met depression remission criteria. We found that IT-TMS could restore the abnormal functional network connectivity between default mode network and precuneus network, left executive control network and sensory-motor network. Furthermore, the changes in functional network connectivity between the default mode network and precuneus network were associated with suicidal ideation, and depressive symptoms were related to connectivity between left executive control network and sensory-motor network. These findings illustrate that IT-TMS is an effective protocol for the accurate restoration of impaired brain networks, which is consistent with clinical symptoms.

## Introduction

Major depressive disorder (MDD) is the most common mental disorder, with 58% of MDD patients having suicidal ideation (SI) and 15% having attempted suicide (1, 2). Although suicide prevention has been thoroughly researched, suicide remains a major cause of morbidity and mortality worldwide (3). The World Health Organization (WHO) reports that approximately 800,000 suicides occur annually across the globe (4), which is a heavy economic burden (5). Only a few treatment options are available for suicidal ideation (e.g., lithium, Emission Computed Tomography) and are only partially effective (6). Individual target-transcranial magnetic stimulation (IT-TMS) represents an original tool that opens new avenues in the treatment of mental disorders, especially MDD (7). However, the clinical responses and neural networks of patients with MDD and SI are unclear.

Brain neural networks are complex systems, and multiple connective networks serve different functions (8, 9). Each functional network controls several brain regions with similar patterns of blood oxygen level-dependent (BOLD) signal changes, whereas each network shows distinct patterns (10–12). In recent years, few studies have investigated the functional network connectivity (FNC) of MDD patients with SI and reported inconsistent results regarding the brain regions associated with resting-state functional alterations (13). Kim et al. researched MDD and MDD patients with SI; FNC between the left superior frontal gyrus and left/right caudate, left superior frontal gyrus and right putamen, left thalamus, left middle frontal gyrus, right thalamus, left postcentral gyrus, etc. were significantly reduced (14). Jung et al. compared the resting-state brain network of MDD patients with suicidal attempts and Health Controls (HCs), and found decreased functional connectivity between the insular network (IN) and default mode network (DMN), as well as the medial prefrontal cortex network (mPFCN) and left frontal-parietal network (LFPN), and increased connectivity between the IN and basal ganglia network (BGN) (15). Chase et al. found that the lower connectivity between the salience network (especially in dACC) and DMN (specifically, dorsal and ventral posterior cingulate cortex) in depression patients with suicidal ideation (16). Although various rest state function Magnetic Resonance Imaging (rsfMRI) studies have revealed functional alterations in brain regions and networks, the network restoration mechanism of IT-TMS rapid action in MDD patients with SI is not yet known.

Traditional TMS spans six consecutive weeks and meets approximately 32% remission and 49% response in depression (17, 18). Stanford accelerated intelligent neuromodulation therapy (SAINT) is a new TMS protocol that is accelerated, safe, tolerable, high-dose, durable, and effective for MDD (19). The core mechanism was subgenual anterior cingulate cortex (sgACC) observed to be hyperactive and its activity was decreased through indirect functional connectivity from the left dorsolateral prefrontal cortex (L-DLPFC) (19–21). However, the L-DLPFC is a large brain area that consists of numerous subunits, which are correlated and some anticorrelated with the sgACC (22). Complex algorithms and precise stimulation of individual anticorrelation subunits have greatly hampered the clinical application of TMS.

**Figure 1:**
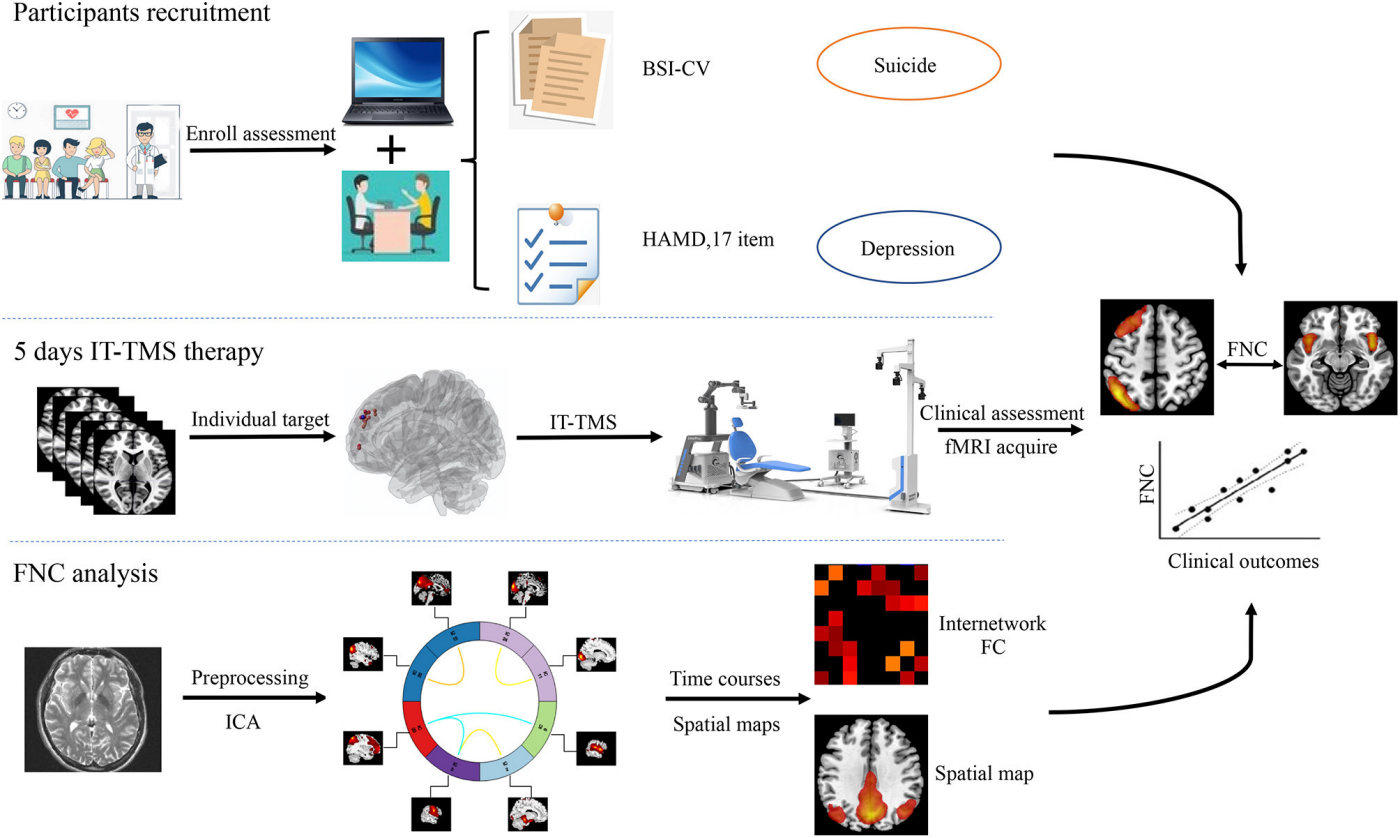
Flowchart of this study design. IT-TMS, Individual Target-Transcranial Magnetic Stimulation; FNC, Functional Network Connectivity; fMRI, Functional Magnetic Resonance Imaging; ICA, Independent Component Analysis; BSI-CV, Beck Scale for Suicidal Ideation—Chinese Version.

In the current study, we try to elucidate intrinsic brain activity and connectivity in MDD patients with SI and HCs by ICA. Firstly, we hypothesized that compared with HCs, depressed patients with suicidal ideation show altered patterns of neural activity, and individual target was generated for the guide intelligent neural navigation system over rsfMRI analysis. Then, the abnormal FNC could repaired after IT-TMS therapy. Finally, we attempted to identify the distinct associated changes in the FNC and clinical scales that assess SI and MDD. A systematic flowchart of the study design is shown in Figure 1.

## Materials and Methods

### Participants

Participants were recruited from the Psychiatric Department of Xijing Hospital (Shaanxi, China) between June 2020 and March 2021. All MDD patients were diagnosed with SI using a structured clinical interview from the Diagnostic and Statistical Manual of Mental Disorder (DSM-5). At the time of screening, our enrollment criteria for MDD patients with SI were as follows: (i) right handed; (ii) ages 18–60; (iii) 17-item Hamilton Depression Rating Scale (HAMD) score >17 and Beck Scale for Suicide Ideation-Chinese Version (BSI-CV) score >6; (iv) a nonpsychotic; (v) a negative urine drug screen, and a negative urine pregnancy test if female; (vi) acute suicide behavior (who needed immediate treatment) were excluded by clinical diagnosis and evaluation (vii) no contraindications of TMS and MRI measurement, such as neurological and psychiatric diseases, history of epilepsy, a brain disorder or abnormality, head trauma, metal or electronic instruments (e.g., intracranial metal device, cochlear implants, cardiac pacemakers, and stents) in the body (23–25). For safety, all MDD patients with SI need to take venlafaxine or duloxetine (serotonin and noradrenaline reuptake inhibitor antidepressant) during this study.

Fifteen patients (ages 18–60, 13 females, mean 25.8) and 15 HCs (ages 18–60, 12 females, mean 32.2) were recruited for this study. And the two groups were not statistically significant in sex (*p* = 0.62), age (*p* = 0.21), and education years (*p* = 0.07). None of the HCs currently or previously had psychopathology, and the above exclusion criteria were also applied to them. The protocol of this study was approved by the Xijing Hospital and the procedures were per the Declaration of Helsinki. All of the participants signed an informed consent form before they participated in this study.

### MRI Acquisition and Data Processing

MRI scans were done using a 3.0 T-UNITED Discovery 770 scanner equipped with a 32-channel head coil. Earplugs were used to reduce the scanner noise, tight but comfortable enough to reduce the head motion. T1-weighted sagittal anatomical images were obtained. The parameters were: sagittal slices = 192; repetition time (TR) = 7.24 ms; echo time (TE) = 3.10 ms; slice thickness/gap = 0.5/0 mm; in-plane resolution = 512 × 512; inversion time (TI) = 750 ms; flip angle = 10°; field of view (FOV) = 256 × 256 mm; voxel size = 0.5 × 0.5 × 1 mm. Resting-state fMRI data were acquired using T2-weighted oblique slices aligned to the anterior and posterior commissure, and the parameters were as follows: sagittal slices = 8,400; repetition time (TR) = 2,000 ms; echo time (TE) = 30 ms; slice thickness/gap = 4/0 mm; in-plane resolution = 64 × 64; inversion time (TI) = 1,100 ms; flip angle = 90°; FOV = 224 × 224 mm; voxel size = 3.5 × 3.5 × 4.0 mm^3^. During the scan, all participants were instructed to keep their eyes closed, relax, think of nothing in particular, but not fall asleep.

As described in previous work across several independent samples, structure and resting-state BOLD data were preprocessed using REST software (26, 27). First, the initial 10 volumes for each participant were discarded to avoid scanning noise, and the remaining 230 volumes were corrected for the acquisition time delay between slices. Then, realigning was used to correct the head motion (<2 mm or 2°) between the time points. Four patients' fMRI data were excluded because of heavy head motion. The effects of nuisance signals and head motion (Friston-24 model) were also regressed out. In the normalization steps, individual structural images were first co-registered with the functional images, segmented, and normalized to the Montreal Neurological Institute (MNI) space using diffeomorphic anatomical registration through the exponentiated lie algebra (28, 29). Finally, the normalized images were smoothed and band-pass filtered (0.01–0.08 Hz).

### Individual Target Generation

**Figure 2:**
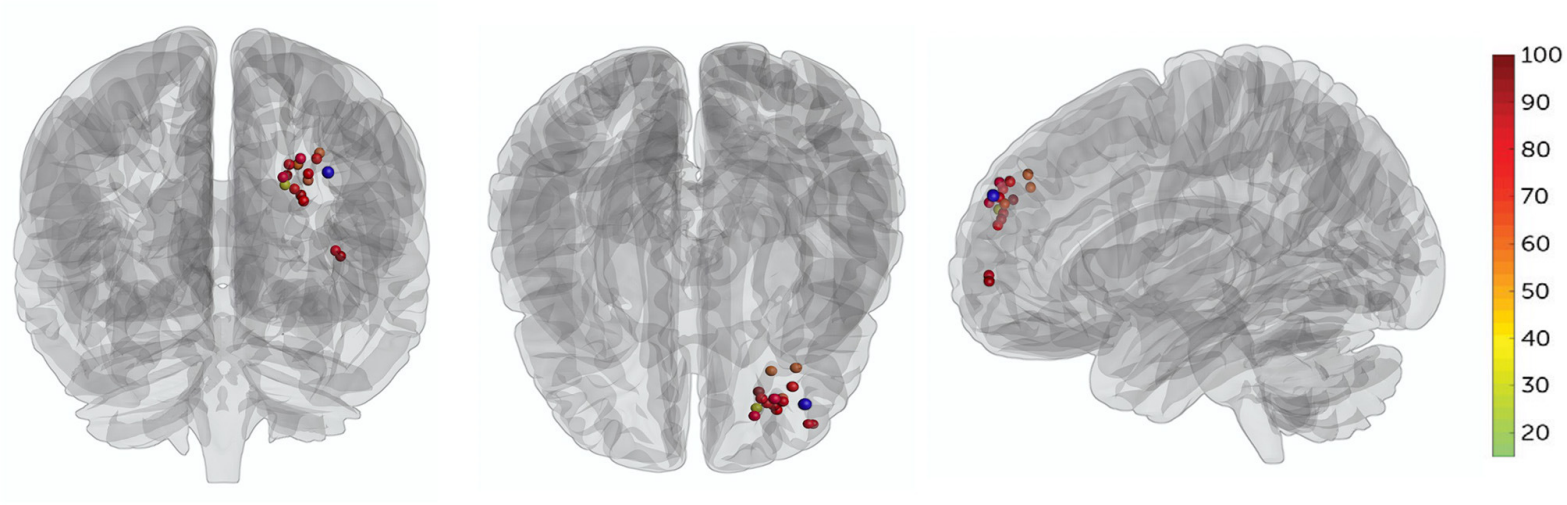
All patients' target locations for IT-TMS therapy. The colors of the targets represent the percentage change in BSI-CV (Beck Scale for Suicidal Ideation—Chinese Version) score after a 5-day therapy, dark red indicating great changes. The F3 location (MNI coordinates−35.3, 49.4, 32.4) is shown in blue.

Patients' targets in the L-DLPFC were set, in which the TMS coil placement was delivered by two separate algorithms (19, 30). First, each patient's L-DLPFC and sgACC were subdivided into numerous functional subunits using a hierarchical agglomerative clustering algorithm. Then, the functional subunits were defined as all voxel pairs being correlated with each other by Spearman's correlation coefficient, and rho ≥0.5. For each functional subunit in the L-DLPFC and sgACC, a single time series value was used to find the single voxel time series, most correlated with the median time series. Once a single time series was identified, Spearman correlation coefficients were used to calculate the correlation matrix between the L-DLPFC and sgACC subunits. Finally, the decision-making algorithm was used to selected most anticorrelation subunit (effective to depression and suicidal symptom), larger size of subunit (easier to target with the IT-TMS coil), and higher spatial concentration (more clustered about the voxels). These three factors are equally weighted for generated individual target, and the numerous individual target location were see in Figure 2.

### Precision Therapy by IT-TMS

The IT-TMS used in this study was the Black Dolphin Navigation Robot (S-50, China), a sub-millimeter smart system that ensures the same subunit stimulation for repeat therapy. The 3D individual mask was beneficial for locating the subunits in the L-DLPFC. Fifty intermittent theta-burst stimulation (iTBS) sessions (1,800 pulses per session, 50-min intervals) were delivered in 10 daily sessions over 5 consecutive days at a 90% resting motor threshold.

### Clinical Assessment and Analysis

Suicidal ideation and depression symptoms were assessed by BSI-CV and a 17-item HAMD scale before and after IT-TMS therapy. Response and remission of suicidal ideation were defined as a reduction of more than 50% compared to the baseline score, and the BSI-CV score was 0 (19). Depression response was defined as a reduction of more than 50% compared to the baseline score, and remission was defined as a Montgomery Asberg Depression Rating Score <11 (31). A neuropsychological test battery was used to assess any neurocognitive side effects after IT-TMS.

SPSS software (version 26) was used for statistical analysis, multiple comparisons were corrected by false discovery rate (FDR) with a corrected significance level of *p* = 0.05 (32). The floor effect of IT-TMS therapy was observed across all clinical scales, and the Shapiro-Wilk residuals of the initial linear mixed model were not normally distributed. Thus, changes in clinical scores were analyzed with a general linear model that used a Satterthwaite approximation of degree of freedom, compound symmetry covariance structure, and robust coefficients excluding violations. All *post-hoc* pairwise comparisons were Bonferroni-corrected.

### Defining Functional Networks Using Independent Component Analysis

Group independent component analysis (ICA) was performed to parcellate the preprocessed fMRI data using the GIFT toolbox (33), the number of independent components was set to 25 by automatic estimation. To ensure estimation stability, the infomax algorithm was repeated 20 times in ICASSO (software for investigating the reliability of ICA estimates by clustering and visualization), and the most central run was selected and analyzed further (34). Finally, the time courses and spatial maps of the patients were obtained by reconstruction. Based on the peak activations in gray matter, we focused on the subdivisions of 11 components maybe correlated with the SI in MDD and defined as resting-state brain networks (RSNs). **Figure 4** showed the spatial map of each component.

Our primary aim was to detect the neural differences between HCs and patients (MDD patients with SI) before and after IT-TMS therapy. The resulting whole-brain maps were threshold at *p* < 0.005, and the voxel level with FDR corrected of *p* < 0.05, cluster size >100. Before the function network connectivity analysis, a serous of post-processing steps including de-trending linear, quadratic, cubic trends and de-spiking detected outliers were performed. Then cut off the frequency of 0.15 HZ and transforming to *z*-score by Fisher's.

For the selected RSNs, two outcomes from the ICA analysis were compared using MANCOVAN (tools for multivariate analysis) with covariates including age and head motion, and the mean temporal correlation across all voxels followed by *post-hoc t*-test (*p* < 0.05). For the outcome measures were correlated with clinical rating score (BSI-CV and HAMD) using Pearson's correlations (*P*_FDR−corrected_ < 0.05).

## Results

### Suicidal Ideation

**Figure 3:**
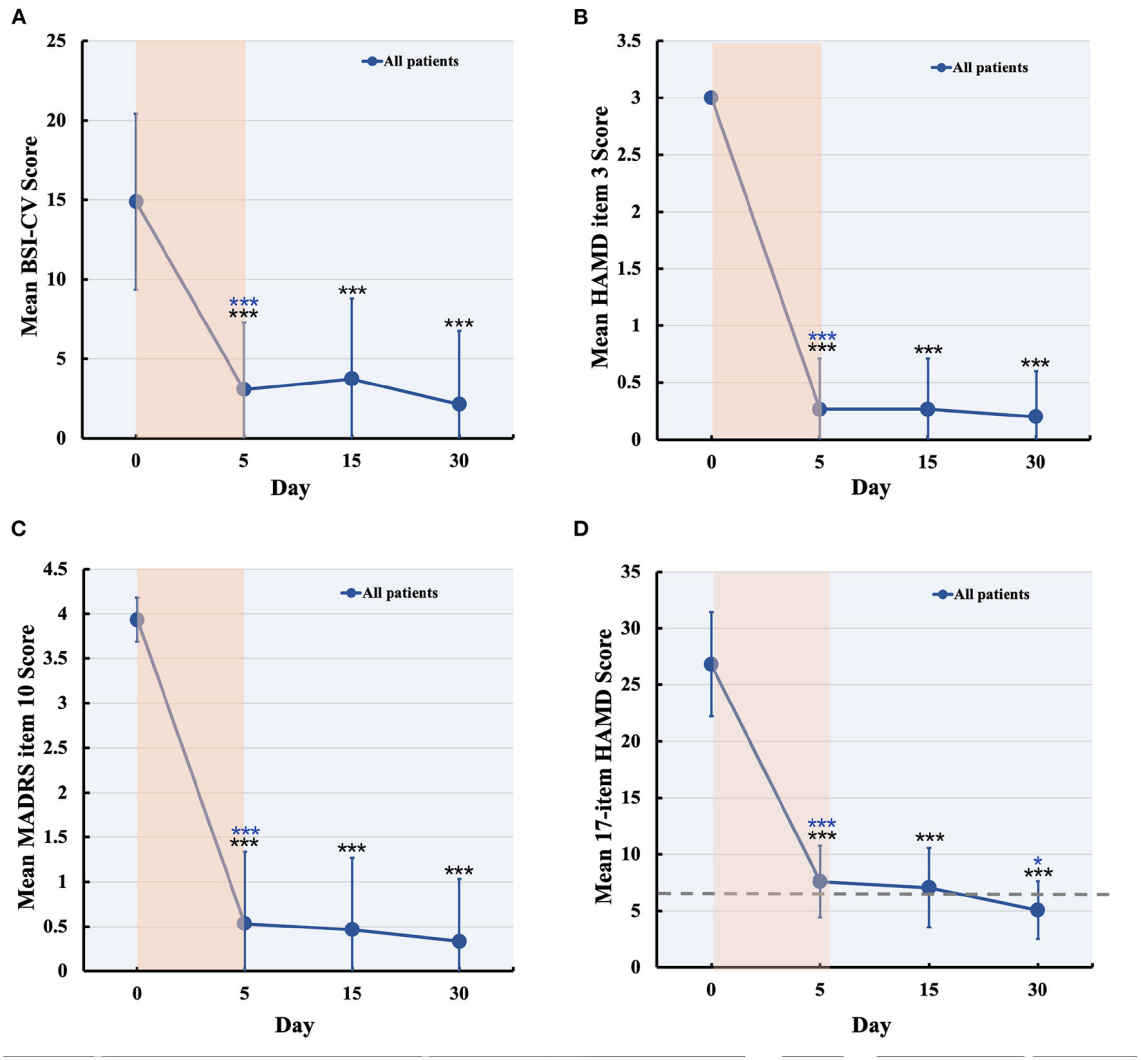
Changes in suicidal and depression score during and after IT-TMS therapy for MDD patients with SI. Panel **(A–C)** shows the suicidal assessment scale of BSI-CV, item 3 of HAMD, and item 10 of MADRS, for patients' baseline (day 0), just post-therapy (5 days), 15, and 30 days post IT-TMS therapy. Panel **(D)** was the mean score of the 17-item HAMD for all patients at baseline (day 0), just post-therapy (5 days), 15, and 30 days post IT-TMS therapy. Black asterisks, significance is compared with baseline; blue asterisks, significance is compared with previous time point. BSI-CV, Beck Scale for Suicide Ideation-Chinese Version; HAMD, Hamilton Depression Rating Score; MADRS, Montgomery–Asberg Depression Rating Scale.

No serious adverse events occurred and no participant dropped out. Two patients experienced scalp numbness and slight pain in the regions of stimulation during IT-TMS therapy. All 15 patients had suicidal ideation at the time of screening for BSI-CV (score >6), and the scores were more than zero on item 3 of the HAMD and item 10 of the MADRS. Changes in suicidal scale scores were assessed using a general linear model with repeated measurement. Figures 3A–C show the baseline score and results after IT-TMS therapy, significant reduction in BSI-CV (F = 38.77, d*f* = 3, *p* < 0.001), item 3 of HAMD (*F* = 296.66, d*f* = 3, *p* < 0.001), and item 10 of MADRS (*F* = 153.72, d*f* = 3, *p* < 0.001). Thirteen patients (86.67%) met a response in suicidal ideation, and eight patients (53.33%) were in remission on the BSI-CV scale just after 5 days therapy. Fifteen days post-IT-TMS met 80% response and 40% remission. Furthermore, the response and remission rates reached 93.33 and 80%, respectively, 30 days after IT-TMS therapy (Table 1). A lower efficacy patient (green dot in Figure 2) after 5 days therapy was improved gradually, and arrived the suicidal remission criteria after 30 days post-therapy.

**Table 1:** Clinical assessment score for patients^a^.

**Measure**	**Baseline**		**Immediately post IT-TMS therapy**			**15 days post IT-TMS therapy**			**30days post IT-TMS therapy**		
	***N***	**Mean (SD)**	**Mean (SD)**	**Response (%)**	**Remission (%)**	**Mean (SD)**	**Response (%)**	**Remission (%)**	**Mean (SD)**	**Response (%)**	**Remission (%)**
**Suicidal ideation**											
BSI-CV	15	14.8 (5.73)	3.06 (4.35)	86.67	53.33	3.73 (5.24)	80.00	40.00	2.13 (4.77)	93.33	46.67
HAMD, item 3	15	3 (0)	0.26 (0.45)	100.00	73.33	0.26 (0.46)	100.00	73.33	0.20 (0.41)	100.00	80.00
MADRS, item 10	15	3.93 (0.26)	0.53 (0.83)	100.00	66.67	0.47 (0.83)	100.00	73.33	0.33 (0.72)	100.00	80.00
**Depression symptoms**											
HAMD, 17 items	15	26.8 (4.75)	7.6 (3.29)	93.33	46.67	7.07 (3.63)	100.00	53.33	5.07 (2.63)	100.00	80.00

a *Response was defined as a reduction of 50% in score compared to baseline; Remission was defined as a score of zero on the suicidal ideation, and a score <7 on 17 item HAMD. SD, standard deviation; IT-TMS, Individual Target-Transcranial Magnetic Stimulation; BSI-CV, Beck Scale for Suicide Ideation-Chinese Version; HAMD, Hamilton Depression Rating Scale; MADRS, Montgomery–Asberg Depression Rating Scale*.

### Depression Symptoms

Significant changes in the 17-item HAMD (*F* = 143.54, d*f* = 3, *p* < 0.001) by general linear model analysis (Figure 3D), and the mean score of all patients was nearly in remission during the 5-day therapy. These scores also steadily decreased after 15 and 30 days, respectively. Finally, we tracked the data at 30 days after therapy and found that the response rate was 100% and remission was 80.00%, which is more efficient in depression. We collected the data of 15 patients after 60 days of IT-TMS therapy, most of them (13 patients, 86.67%) were not depression or suicidal symptom except for two patients. The two patients who relapsed maybe required more days of treatment to achieve the remission criterion, and another 5 days therapy were delivered.

### Brain Network Activity

**Figure 4:**
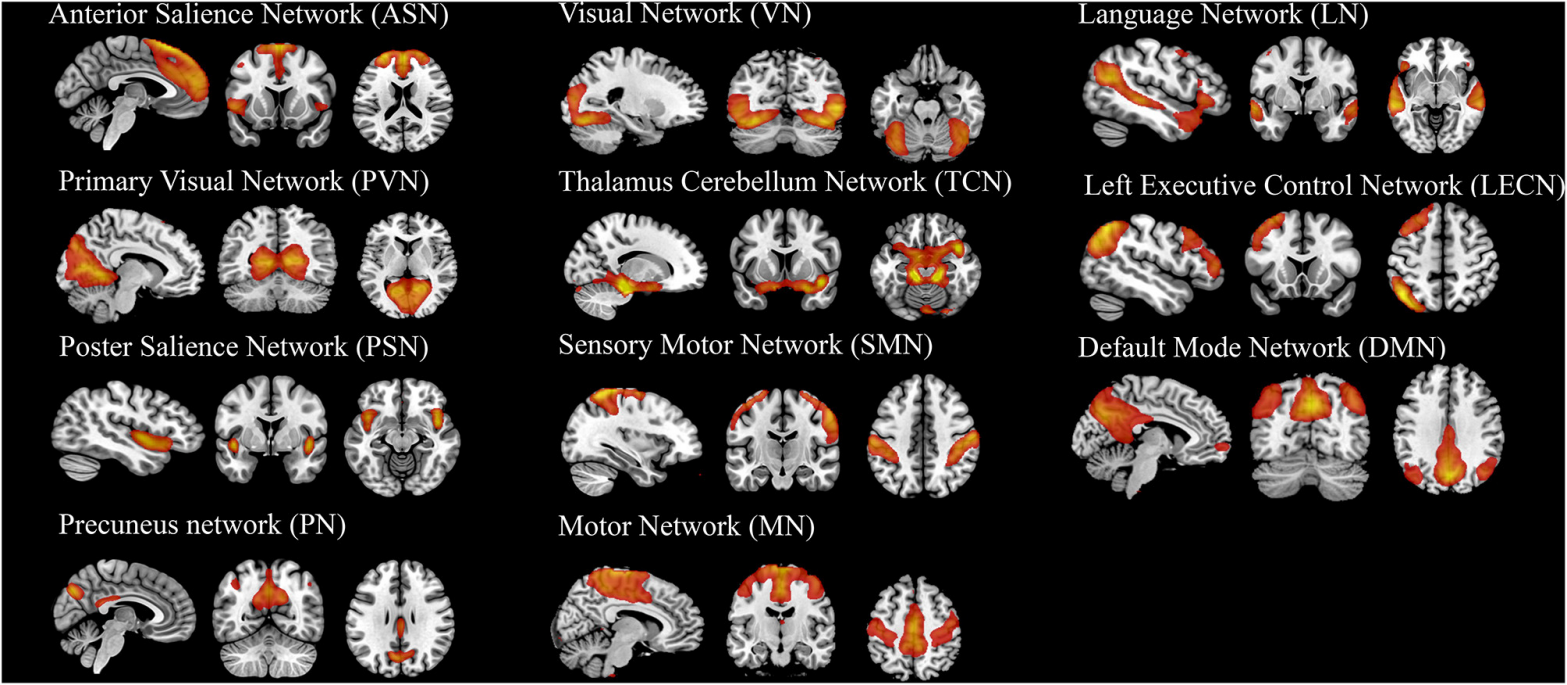
The spatial map of 11 components (RSNs) derived from group ICA analysis. The statistical threshold was set at *T* > 20.

Twenty-five components estimated by ICA and 11 temporal coherent signals confined to the brain were selected as the RSNs. Here, we labeled the RSNs according to their spatial locations or a previous study described (Figure 4 and Supplementary Table S1) show the spatial map of RSNs: anterior salience network (ASN), primary visual network (PVN), posterior salience network (PSN), precuneus network (PN), visual network (VN), thalamus cerebellum network (TCN), sensory-motor network (SMN), motor network (MN), language network (LN), left executive control network (LECN), and default mode network (DMN).

### Functional Network Connectivity

**Figure 5:**
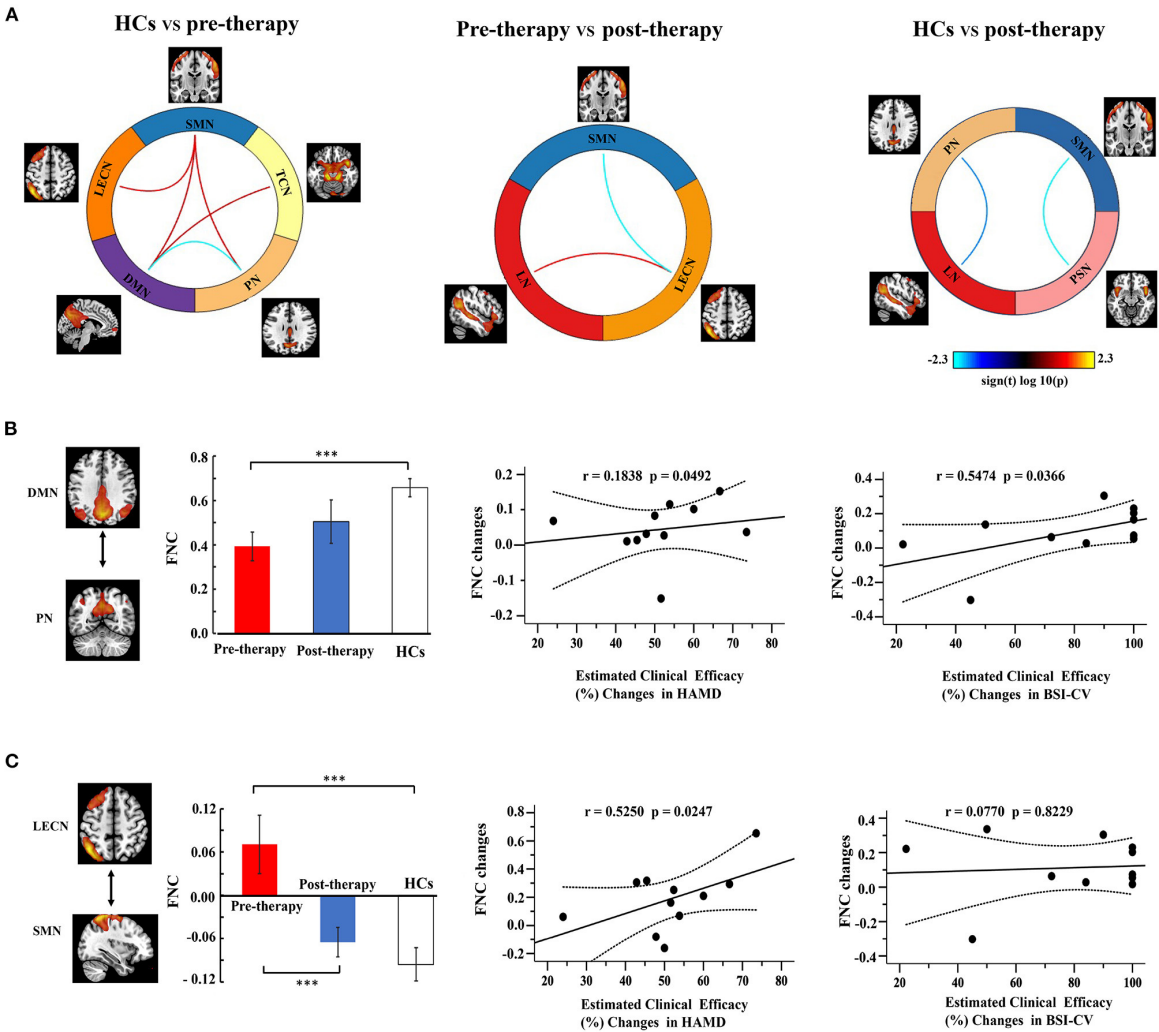
Function network connectivity (FNC) between RSNs. **(A)** FNC differences in the selected FNC. The blue line indicates a decreased FNC and the red lines indicates improved FNC (*P* < 0.05). **(B,C)** shows the relationships between the FNC and clinical efficacy. Error bars indicate the standard error. Dotted lines indicated the significantly changes of groups. ^*^p < 0.05. ^**^p < 0.01. ^***^p < 0.001.

Figure 5A shows a significant difference of three groups on FNC between 11 RSNs, and these results performed *post-hoc* tests. Compared to the HC group, MDD patients with SI exhibited a decreased FNC between DMN and PN, as well as an enhanced FNC in DMN and TCN, DMN and SMN, LECN and SMN, SMN, and PN. Compared to the pre-therapy and post-therapy images, FNC was decreased in SMN and LECN, but increased in LECN and LN. Finally, we explored the post-therapy and HCs and found that the FNC between PSN and SMN, PN and LN were significantly decreased.

Correlation analysis was used to investigate the relationship between FNC and clinical efficacy (Figures 5B,C). The FNC between the DMN and PN gradually increased from the pre-therapy (MDD patients with SI) to the post-therapy and from the post-therapy groups to the HCs. The FNC changes in DMN-PN were positively correlated with the 17 item HAMD score (*r* = 0.1838, *P*_FDR−corrected_ = 0.0492) and BSI-CV scores (*r* = 0.5474, *P*_FDR−corrected_ = 0.0366). Furthermore, the FNC in LECN and SMN showed a significant correlation with HAMD (*r* = 0.5250, *P*_FDR−corrected_ = 0.0247), but not with BSI-CV. No other correlations were statistically significant between the HCs, pre-therapy, and post-therapy.

## Discussion

IT-TMS is a non-invasive, safe, and effective method for MDD patients with SI (6), but there are only a few systematic research on the FNC mechanism. Here, we found that IT-TMS significantly decreased suicidal ideation and depressive symptoms within five continuous days. Furthermore, suicidal ideation and depressive symptoms were not recurrent 1 month later, and the depression remission rate (80%) of 17 item HAMD was higher than that of SAINT protocol (65%) (19). These differences between the observed and SAINT protocols may be due to the sub-millimeter smart navigate system that ensures the same subunit stimulation for repeat therapy.

Recently, Northoff reported that various depressive and suicidal symptoms can be translated as intrinsic brain activity in spatiotemporal disturbances, which is the organization of the resting-state network activity (35). We found that MDD patients with SI had several significant changes in functional network connections, including LECN-SMN, DMN-SMN, DMN-TCN, SMN-PN, and DMN-PN, compared to HCs. Specifically, DMN and PN showed decreased functional connectivity, but the others improved. These results are compatible with a recent view on the heterogeneity connection of the DMN, ECN, and SMN, which can be reorganized according to specific demands by functional anatomic fractionation (36, 37). Our results illustrate the complex network imbalance derived from deficits in suicidal ideation and depressive symptoms.

Based on previous research, Serafini et al. proposed that frontal limbic or frontal-parietal cerebellar pathways and DMN connection abnormalities increase emotional dysregulation (self-focus, hopelessness, and suicidal ideation) (13). These results consistent with our study that the functional network connection of MDD patients with SI were significantly changes, especially in DMN, LECN, SMN, TCN, and PN. Moreover, MDD patients with SI showed more alterations in the SMN, LECN, and LN through IT-TMS therapy: increased functional connectivity in LECN-LN and decreased functional connectivity in LECN-SMN. Decreased connections in limbic regions may contribute to suicidal ideation regulation by emotion processing (38, 39), whereas increased connections in the executive and language networks may contribute to depressive symptoms (40). Here, we speculated that the brain responses of MDD patients with SI represent an insufficient compensatory mechanism for network interfering activity, which perhaps modulation by the IT-TMS therapy.

Mechanistic research further revealed that functional connectivity changes in various brain networks are significantly related to suicidal ideation and depression symptoms. It is noteworthy that the core rest-state networks of the DMN and PN decreased connections, suggesting an intimate link to suicidal ideation (41–43). A previous study in MDD patients with SI has also reported that the DMN and PN positive connection benefits depression and suicidal remission (44). These two networks play a key role in emotion and cognitive modulation and show a bias toward self-centered rather than self-other processing (41, 45).

Interestingly, IT-TMN therapy significantly improved patients' anti-connectivity between LECN and SMN, which is related to higher 17 item HAMD score changes. First, the LECN engages in cognitive control to support goal-directed behavior, consistent with depression and suicidal patients' difficulty in regulating emotions (46). Our finding of decreased positive connection between LECN and SMN by IT-TMN was consistent with the evidence for increased functional connectivity in MDD patients (47). The current findings not only may add important literature to potential neurobiological mechanisms, but also have high clinical relevance for exposing the MDD patients with SI neuro-biomarkers.

## Limitations and Conclusion

This study has several limitations. First, the small sample size and the age might not be representative of the general population. Further research with larger sample size and a broader age range may validate our findings. Second, although we identified a meaningful functional network from a range of ICA-derived components through a structural selection procedure, this may have influenced our interpretation. Third, rsfMRI data can be related to the effects of brain activity, but we did not ensure that participants thought of nothing in particular. Finally, the sham stimulation group and only drug group were also required to exclude false-positive results.

To the best of our knowledge, this study is the first to examine multiple brain network interactions after IT-TMS therapy for MDD patients with SI. We demonstrated that widespread but discrete network changes in functional networks and their abnormalities are associated with clinical efficiency. Moreover, DMN-PN and LECN-SMN functional connectivity may act as mediators of suicidal ideation or depressive symptoms. These findings might expand existing knowledge concerning suicidal and depression function network organization. More generally, they may ultimately inform a clinical protocol for the remission of suicidal ideation and depressive symptoms by IT-TMS.

## Data Availability Statement

The original contributions presented in the study are included in the article/Supplementary Material, further inquiries can be directed to the corresponding author/s.

## Ethics Statement

The studies involving human participants were reviewed and approved by Medical Ethics Committee of the First Affiliated Hospital of PLA Air Force Military Medical University. The patients/participants provided their written informed consent to participate in this study.

## Author Contributions

HW, SQ, and SL designed the current study. NT, JL, and YC collected the data. XL, LS, and YR analyzed the data. CS and YW wrote the manuscript. All authors read and approved the final manuscript.

## Funding

This study was financially supported by the National Natural Science Foundation (number 81974215) and Social Development Area in Shaanxi Key Projects (number 2017ZDXM-SF-047).

## Conflict of Interest

The authors declare that the research was conducted in the absence of any commercial or financial relationships that could be construed as a potential conflict of interest.

## Publisher's Note

All claims expressed in this article are solely those of the authors and do not necessarily represent those of their affiliated organizations, or those of the publisher, the editors and the reviewers. Any product that may be evaluated in this article, or claim that may be made by its manufacturer, is not guaranteed or endorsed by the publisher.
