# Supplementary material for: Clinical Response of Major Depressive Disorder Patients With Suicidal Ideation to Individual Target-Transcranial Magnetic Stimulation
Source: Front Psychiatry. 2021 Nov 5;12:768819. doi: 10.3389/fpsyt.2021.768819 (PMC8602581; doi:10.3389/fpsyt.2021.768819)
Supplement: Supplementary file 1 [file Table_1.DOCX]

Table S1. Summary of RSNs.

| RSN | Region | cluster | x | y | z |
| --- | --- | --- | --- | --- | --- |
| ASN | L Insula  R Insula  Anterior Cingulate cortex | 15025  14128  9288 | -38  45  0 | 18  19  33 | -9  -9  11 |
| PVN | L Calcarine gyrus | 17390 | -12 | -60 | 6 |
|  | R Calcarine gyrus | 14885 | 11 | -62 | 8 |
| PSN | L Insular  R Insular | 15025  14128 | -46  46 | 8  2 | 6  6 |
|  | L Cuneus | 12576 | 2 | -74 | 26 |
| PN | R Precuneus | 4632 | 8 | -44 | 20 |
|  | L Angular  R Angular | 3104  768 | -36  38 | -56  -58 | 36  40 |
| VN | R Inferior occipital gyrus  L Inferior occipital gyrus  R Fusiform gyrus  L Fusiform gyrus | 7664  7409  15559  14844 | 42  -44  28  -26 | -78  -78  -78  -78 | 0  -2  -2  -5 |
| TCN | R Cerebellum  L Cerebellum  Thalamus | 15584  15291  8399 | 12  -13  13 | -33  -35  -25 | -18  -16  3 |
| SMN | R Postcentral gyrus | 28262 | 45 | -36 | 60 |
|  | L Postcentral gyrus | 28922 | -46 | -37 | 58 |
| MN | L Precentral gyrus  R Precentral gyrus  L Supplementary motor area  R Supplementary motor area | 34118  32613  15636  17655 | -23  23  -4  12 | -29  -28  -11  -13 | 72  72  72  73 |
| LN | L Middle temporal gyrus | 37521 | -54 | -62 | 20 |
|  | R Middle temporal gyrus | 34163 | 52 | -54 | 20 |
|  | L Precuneus | 27545 | -4 | -54 | 33 |
|  | L Middle Temporal pole | 3292 | -39 | 17 | -23 |
|  | R Middle Temporal pole | 6137 | 56 | 8 | -22 |
|  | L Inferior Frontal Gyrus | 19382 | -45 | 28 | -5 |
|  | R Inferior Frontal Gyrus | 15682 | 55 | 28 | 7 |
|  | L Superior Frontal Gyrus | 26495 | -14 | 28 | 53 |
| LECN | L Inferior Frontal Gyrus  L Inferior Parietal  L Middle temporal gyrus | 19672  19382  37521 | -53  -42  -57 | 16  -63  -46 | 26  54  -7 |
| DMN | R Cuneus | 11323 | 7 | -74 | 36 |
|  | L Cuneus | 11949 | -2 | -77 | 36 |
|  | L Angular gyrus | 9313 | -44 | -65 | 36 |
|  | R Angular gyrus | 13998 | 49 | -62 | 37 |
|  | L Middle frontal cortex | 36381 | -10 | 56 | -7 |
